# Identification of DNA N^6^-methyladenine sites by integration of sequence features

**DOI:** 10.1186/s13072-020-00330-2

**Published:** 2020-02-24

**Authors:** Hao-Tian Wang, Fu-Hui Xiao, Gong-Hua Li, Qing-Peng Kong

**Affiliations:** 1 State Key Laboratory of Genetic Resources and Evolution/Key Laboratory of Healthy Aging Research of Yunnan Province, Kunming Institute of Zoology, Chinese Academy of Sciences, Kunming, 650223 China; 2 Center for Excellence in Animal Evolution and Genetics, Chinese Academy of Sciences, Kunming, 650223 China; 3 Kunming Key Laboratory of Healthy Aging Study, Kunming, 650223 China; 4 KIZ/CUHK Joint Laboratory of Bioresources and Molecular Research in Common Diseases, Kunming, 650223 China; 5 Kunming College of Life Science, University of Chinese Academy of Sciences, Beijing, 100049 China

**Keywords:** DNA N^6^-methyladenine, Machine learning, XGBoost

## Abstract

**Background:**

An increasing number of nucleic acid modifications have been profiled with the development of sequencing technologies. DNA N^6^-methyladenine (6mA), which is a prevalent epigenetic modification, plays important roles in a series of biological processes. So far, identification of DNA 6mA relies primarily on time-consuming and expensive experimental approaches. However, in silico methods can be implemented to conduct preliminary screening to save experimental resources and time, especially given the rapid accumulation of sequencing data.

**Results:**

In this study, we constructed a 6mA predictor, p6mA, from a series of sequence-based features, including physicochemical properties, position-specific triple-nucleotide propensity (PSTNP), and electron–ion interaction pseudopotential (EIIP). We performed maximum relevance maximum distance (MRMD) analysis to select key features and used the Extreme Gradient Boosting (XGBoost) algorithm to build our predictor. Results demonstrated that p6mA outperformed other existing predictors using different datasets.

**Conclusions:**

p6mA can predict the methylation status of DNA adenines, using only sequence files. It may be used as a tool to help the study of 6mA distribution pattern. Users can download it from https://github.com/Konglab404/p6mA.

## Background

DNA N^6^-methyladenine (6mA) is an important epigenetic modification of nucleic acid, firstly characterized in bacteria [1]. In contrast to 5mC, 6mA remains poorly studied and was previously thought to only occur in prokaryotes [2]. Accumulating evidences, however, has confirmed that it also exists in eukaryotes, including zoological and botanical species (e.g., *Arabidopsis thaliana*, *Mus musculus*, *Danio rerio*, and *Sus scrofa*). Recently, two studies found that DNA 6mA sites also exist extensively in the genomes of humans [3] and rice [4], thus deepening our understanding of this modification in high-grade organisms.

DNA 6mA plays important roles in various biological processes, such as the restriction–modification system [5, 6], DNA replication and repair [7, 8], nucleoid segregation [9, 10], and transcription [11]. To detect DNA 6mA modification, a series of experimental methods have been developed, such as methylated DNA immunoprecipitation sequencing [12], liquid chromatograph–tandem mass spectrometry [3], capillary electrophoresis and laser-induced fluorescence [13], and single-molecule real-time sequencing (SMRT-seq) [14]. However, these experimental procedures are expensive and time-consuming and thus largely limited its application in DNA 6mA study, urging the necessity for the development of bioinformatics-based approaches to predict methylated adenine sites in genomes.

Machine learning builds models by handling features to perform specific tasks and has been widely applied in biological issues, including post-transcription RNA identification [15–17], promoter discovery [18–20], and nucleotide modification prediction [21–23]. The occurrence of 6mA relies on the properties of its surrounding sequences, which play vital roles in methyltransferase/demethylase-dependent catalytic processes [3, 24, 25]. Recently, some machine learning-based predictors, e.g., iDNA6mA-PseKNC [26] and i6mA-Pred [27], were developed to identify 6mA sites at the genomic level. The former was trained with mouse data and achieved a high recall ratio in several datasets, whereas the latter was designed to predict 6mA sites in rice. To the best of our knowledge, however, there is no 6mA predictor trained on multi-species data.

In this study, we constructed a predictor, p6mA, to identify DNA 6mA sites by sequence-based features. The predictor was trained on dataset from four species: i.e., *Oryza sativa* (rice), *Drosophila melanogaster* (fruit fly), *Caenorhabditis elegans* (worm), and *Homo sapiens* (human). The DNA sequences were transformed into numeric vectors by extracting 172 features. We selected key features using the maximum relevance maximum distance (MRMD) method [28] and constructed the predictor using the Extreme Gradient Boosting (XGBoost) algorithm [29]. Comparison with other existing tools demonstrated that p6mA outperformed other methods in several aspects. Users can download p6mA from https://github.com/Konglab404/p6mA.

## Results

### Nucleotide composition and conservation analysis

**Fig. 1:**
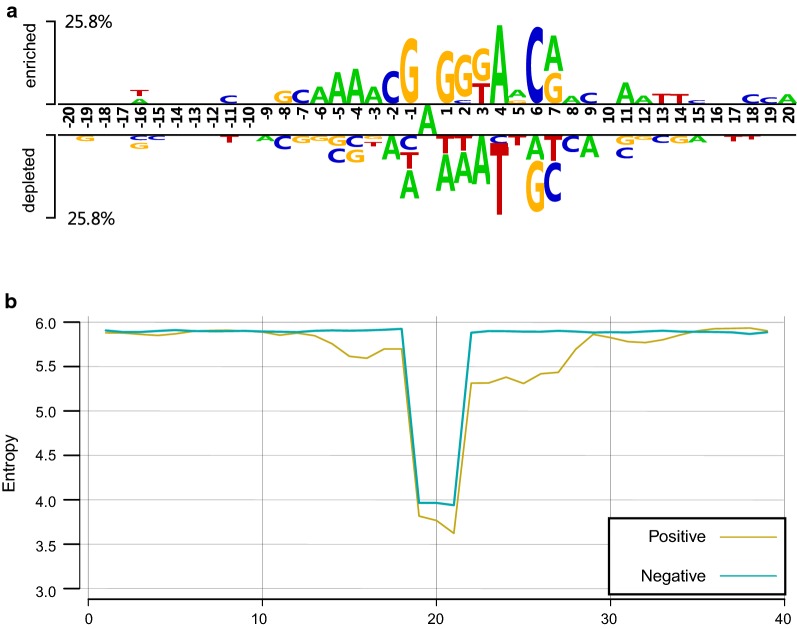
Nucleotide composition of benchmark dataset. **a** Two Sample Logos result of benchmark dataset, top panel denotes the nucleotide enrichment status of 6mA-containing sequences and bottom panel is of non-6mA-containing sequences. **b** Entropy analysis of 6mA- and non-6mA-containing sequence. Red line denote 6mA-containing sequences and blue one denotes non-6mA sequences

In this study, we constructed an aggregated benchmark dataset by four species’ data. There are 3040 positive samples and 3040 negative samples (Table 1). All the samples are 41 nt long with an adenosine (A) in the center. We adopted Two Sample Logos [30] to visualize significantly overrepresented and underrepresented sites with a threshold of *p *< 0.05. The nucleotide enrichment status, as shown in Fig. 1a, showed that there exists nucleotide distribution bias between 6mA and non-6mA containing sequences. For example, in 6mA-containing sequences, GAGG motif was enriched in center and adenosine was enriched in the + 4 nt position. The above results indicated that the surrounding nucleotide composition information can be adopted to discriminate 6mA and non-6mA sites.

**Table 1:** The statistics of benchmark dataset in this study

Dataset	# Positive samples	# Negative samples	Reference genome
*O. sativa*	880	880	MH63
*D. melanogaster*	728	728	dm3
*C. elegans*	632	632	ce10
*H. sapiens*	800	800	hg38
Aggregated	3040	3040	–

Next, we investigated whether sequence bias can cause differences in conservatism. We then performed entropy analysis, aiming to determine trinucleotide-positioned conservatism differences between 6mA and non-6mA sites [31]. The entropy of trinucleotides at each position was calculated as follows:

1 $${\text{Ent}}_{i} = - \mathop \sum \limits_{j - 1}^{n} p(3{\text{mer}}_{j} |i) \cdot \log_{2} p(3{\text{mer}}_{j} |i),$$

where *n* denotes the total number of trinucleotide combinations in the *i*th position and *p*(*3*mer_*j*_*|i*) denotes the frequency of the *j*th trinucleotide at the *i*th position in the positive/negative samples. Two 39-dimensional numerical vectors were generated to express the entropy values at positions of positive and negative samples.

Information entropy was used to evaluate chaos in the signal processing field and help to reflect conservatism [31]. A lower entropy value, which means less chaos, indicates that the site concerned is more conserved. Figure 1b shows the comparison of trinucleotide entropy at different positions in the 6mA and non-6mA samples. Samples with 6mA sites display lower entropy values, especially at center adenine positions, than those with non-6mA sites. Our results showed that the positive samples possessed more conservatism that negative samples in specified positions, especially in regions surrounding center adenine sites.

The Two Sample Logos and entropy analysis results both supported that positioned nucleotide information is able to discriminate between 6mA and non-6mA sites, thus providing a reasonable basis for the application of positioned sequence feature extraction methods like PSTNP.

### Feature selection and parameter tuning

**Fig. 2:**
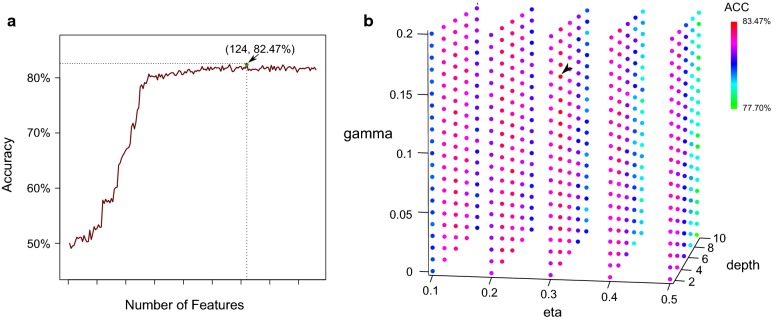
Feature selection and parameters tuning. **a** IFS curve of feature selection. **b** Grid-search results of parameter tuning

We used three methods (i.e., PSTNP, EIIP, and physicochemical properties) to extract features. Each sample was transformed into a 172-dimensional numerical vector, though the feature set also included redundant features. To reduce computational resource waste, we used MRMD score, an index positively related to feature importance, and incremental feature selection (IFS) to select optimal feature sets for each dataset. Features were ranked by MRMD score from highest to lowest. The features from the ranked list were then added one-by-one to a new set and used to construct an XGBoost-based model with default parameters. Model performance was evaluated by tenfold cross-validation and the feature set with highest accuracy was chosen as the optimal set. As shown in Fig. 2a, the highest accuracy (82.47%) was obtained when the optimal 124 features were included. Therefore, we trained the model by its top-ranked 124 features.

We then trained models for each dataset using their optimal feature sets. To obtain better performance, a grid search strategy was used to conduct model tuning. Three parameters (i.e., gamma, eta, and max_depth) of XGBoost were optimized in the spaces [0, 0.2], [0.1, 0.5], and [2, 10] with steps of 0.1, 0.1, and 2, respectively. We trained 525 models and the parameter set with the highest tenfold cross-validation accuracy was chosen as the parameter set of the model. Accuracy scatter plots of the model based on different parameter combinations are shown in Fig. 2b. Results demonstrated that the optimal parameter set was gamma = 0.16, eta = 0.3, and max_depth = 4. Thus, we trained the model using the three optimal parameters. Then we performed jackknife test to evaluate its performance and the accuracy is 82.04%. Accordingly, a predictor named p6mA was implemented.

### Comparison with existing predictors

To evaluate the prediction performance of p6mA, we compared it with three existing predictors, i.e., iDNA6mA-Pred [27], iDNA6mA-PseKNC [26] and MM-6mAPred [32]. The jackknife test result was applied to measure the predictive power of our methods.

**Fig. 3:**
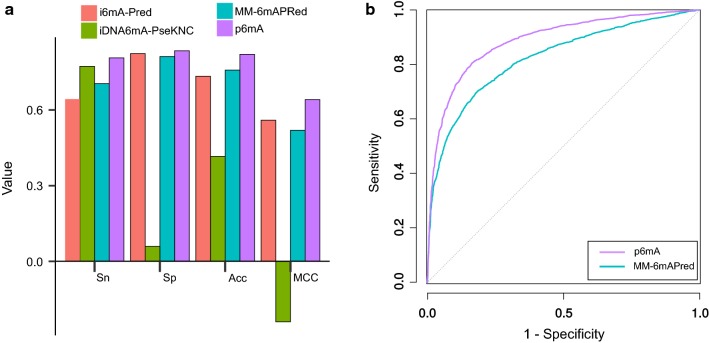
Comparison between p6mA and other existing predictors by benchmark dataset. **a** The performance of the 4 predictors. **b** ROC curves of p6mA and MM-6mAPred

As shown in Fig. 3a, p6mA performed better than the three predictors, it obtained the highest values among the four metrics (i.e., sensitivity, specificity, accuracy, and Matthews Correlation Coefficient). MM-6mAPred has the second highest accuracy (Acc) and second highest Matthews Correlation Coefficient (MCC), while its sensitivity (Sn) is 70.46%, which is ~ 10% lower than that of p6mA. iDNA6mA-PseKNC’s sensitivity is 77.27%, while its specificity is only 5.95%. i6mA-Pred obtains specificity of 82.37%, while the sensitivity is 64.31%. The details of the performances can be found in Additional file 1: Table S1.

MM-6mAPred provides the prediction score for each sample, so we plotted its receiver operating characteristic (ROC) curve and compared it with ROC curve of p6mA (Fig. 3b). The area under the ROC curve (auROC) were calculated, p6mA has a larger auROC (0.8871) than MM-6mAPred (0.824).

### Independent validation on *A. thaliana* dataset

We then performed independent validation on a dataset from another species. As a vital model flowering plant, *A. thaliana* is a good species to test our predictor, with its N^6^-methyladenine modification landscape previously reported in 2015 [33]. The modification data of *A. thaliana* were downloaded from MethSMRT and a dataset for independent validation was constructed. We obtained 1055 non-redundant positive samples and 1055 non-redundant negative samples from the reference genome TAIR10. The dataset construction method was similar to the benchmark dataset.

**Fig. 4:**
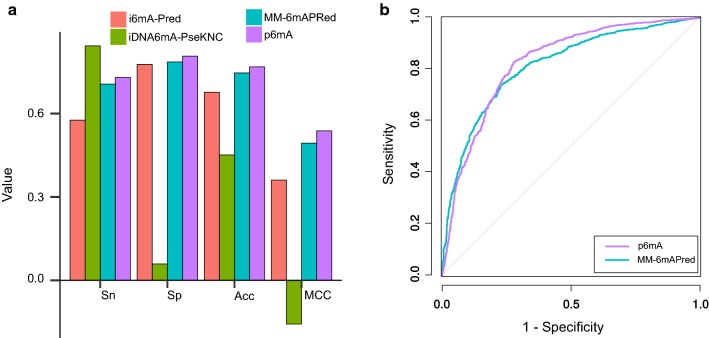
Results of independent validation by *A. thaliana* dataset. **a** The performance of the 4 predictors. **b** ROC curves of p6mA and MM-6mAPred

We compared p6mA with iDNA6mA-Pred, iDNA6mA-PseKNC and MM-6mAPred by the *A. thaliana* dataset. As shown in Fig. 4a, iDNA6mA-PseKNC obtained the highest Sn (84.36%), but performed less well in the other three indicators, especially Sp (5.88%). The p6mA achieved better overall performance in comparison with the other predictors: the highest Sp (80.66%), Acc (76.82%), and MCC (0.5379). We also plotted MM-6mAPred’s ROC curve and compared it with ROC curve of p6mA (Fig. 4b), p6mA has a higher auROC (0.8246) than that of MM-6mAPred (0.8141). Overall, we demonstrated the robustness of p6mA and its superiority over other existing methods by independent validation. The details of the performances can be found in Additional file 1: Table S2.

### Software package introduction

To facilitate the application of our predictor, we implemented p6mA in R language, with the code stored in GitHub (https://github.com/Konglab404/p6mA). The feature extractor methods were also implemented, users just need to provide the input data in fasta format files. Each sequence of the input file should be a 41-bp-length sequence and the center position (e.g., the 21th nucleotide) is the A (adenine) for predict, like:

> human_seq_1101_hg19

ATAGTGTAGTGAGCGTACGTAACGTGAAGTGAGTGAGTAGC.

The output file of p6mA is a text file containing sequence names, scores, and predicted modification status. The detailed usage and installation guide and the example input/output files can also be found on the repository page.

## Discussion

In this study, we built a 6mA predictor p6mA and showed that it is a more robust and competitive 6mA predictor than other existing ones, as determined using benchmark dataset and independent validation. We not only developed a convenient tool for predicting 6mA, but also indicated the portability of the position-based feature extraction method in biological subjects, especially in nucleotide modification prediction. Besides, recent research showed that different species may have different 6mA preference motifs, e.g., the AGAAT motif of *C. elegans* [34]. This phenomenon prompts us that it is necessary to build 6mA predictors by multiple species’ data.

In the independent test by *A. thaliana* data, although p6mA obtained a higher auROC than that of MM-6mAPred, the ROC cures displayed that the specificity of p6mA needs to be improved. Due to the rarity of modified nucleotides in genome, some rare category exploration methods, e.g., RCLens [35], could also be adopted into uncommon nucleic acid modification prediction problems in the future.

As an epigenetic modification, methylation of adenine is a complex biological process that may be affected by other factors, such as chromatin topology and cytoplasmic physicochemical properties. Therefore, we will incorporate additional information to improve the performance of the discrimination ability in the future and different feature extraction methods may be used to construct a more powerful epigenetic modification predictor.

## Conclusions

In summary, we developed a new bioinformatics tool, p6mA, for predicting 6mA-modified sites. We also implemented the predictor as an R software package for ease of use. p6mA was built by multiple species’ data and it may help the investigation of 6mA modification pattern in different species’ genomes.

## Methods

### Benchmark dataset construction

Fruit fly and worm 6mA-positive samples were obtained from the MethSMRT database [36] and human 6mA-positive samples were obtained from the HuaXia1 assembly [37]. To construct a high-quality dataset, 6mA sites with identification Qv scores, which represent the confidence level of a modification, of less than 30 (*p*-values < 0.001) were filtered. We extracted ± 20 nt sequences from the 6mA sites for each sample to a final sequence length of 41 nt. To reduce sequence-homology bias, CD-HIT v4.6.8 [38] was utilized to generate non-redundant sequence sets with an identity threshold of 0.6.

The 6mA-negative samples from the above three species (i.e., fruit fly, worm, and human) were constructed by selecting non-6mA adenines randomly from the reference genomes (hg38, dm3, and ce10). To ensure negative sample quality, the non-6mA sites were not located in the ± 500-bp flanking regions of positive 6mA sites. We also extracted ± 20 nt sequences for each negative site as the negative samples, and sequence identity was also less than 0.6.

880 6mA-positive and 880 6mA-negative samples of rice were obtained from i6mA-Pred (http://lin-group.cn/server/i6mA-Pred) and retrieved by SMRT-seq [4]. All sequences were 41 nt long, with the 6mA site at the center position.

Finally, we constructed benchmark dataset from four species’ data: i.e., rice, fruit fly, worm, and human. The final benchmark dataset contains 3040 positive samples and 3040 negative samples. Each DNA sequence in the study could be simplified as the formation:

2 $${\text{Se}} = N_{1} N_{2} N_{3} \ldots N_{L - 1} N_{L} ,$$

where

3 $$N_{i} \in \left[ {{\text{A}}\left( {\text{adenine}} \right), {\text{C}}\left( {\text{cytosine}} \right), {\text{G}}\left( {\text{guanine}} \right), {\text{T}}\left( {\text{thymine}} \right)} \right]$$

represents the *i*th nucleotide in the sequence. Here, we used the following three sequence-based features: (1) electron–ion interaction pseudopotential (EIIP); (2) position-specific triple-nucleotide propensity (PSTNP), and (3) physicochemical properties. These feature extraction methods were implemented in our in-home R package *RTFE* (https://github.com/ritianjiang/RTFE), the details of which are introduced in the following sections. Briefly, we transformed each sample into a 172-dimensional numerical vector.

### EIIP features

Electron–ion interaction pseudopotential, which reflects the electronic properties of nucleotides, was first used to predict the coding potential of genomic regions [39]. EIIP-based feature extraction methods were then widely applied in field prediction and classification, including the prediction of nucleosome positioning [40] and identification of E-gene signature [41].

The EIIP feature vector was constructed as follows:

4 $$D = \left[ {{\text{EIIP}}_{\text{AAA}} \cdot f_{\text{AAA}} , {\text{EIIP}}_{\text{AAC}} \cdot f_{\text{AAc}} , \cdots {\text{EIIP}}_{\text{TTT}} \cdot f_{\text{TTT}} ,} \right],$$

where EII*P*_*xyz*_ denotes the average EIIP value of three nucleotides (*x*, *y*, and *z*), *f*_*xyz*_ denotes the frequency of the 3-tuple nucleotides *xyz* in the sample sequence and *x, y, z *∈ (A, C, G, T). The EIIP values for the four nucleotides are:

5 $$\left\{ {\begin{array}{*{20}c} {{\text{EIIP}}_{\text{A}} = 0.1260} \\ {{\text{EIIP}}_{\text{C}} = 0.1340} \\ {{\text{EIIP}}_{\text{G}} = 0.0806} \\ {{\text{EIIP}}_{\text{T}} = 0.1335} \\ \end{array} } \right..$$

Using this method, we generated 64 features.

### PSTNP features

Position-specific triple-nucleotide propensity describes the differences in nucleotide composition at each position between the sequences with and without 6mA modification. As a statistics-based feature extraction method, PSTNP has been used to address multiple molecular biological problems, including DNA N^4^-methylcytosine (4mC) site prediction [22], enhancer prediction [42], and σ70 promoter predictor [43].

Two subtypes of PSTNP were used in this study, i.e., single-stranded and double-stranded (PSTNP_SS_ and PSTNP_DS_, respectively). The PSTNP_SS_ features are based on the single-stranded characteristics of DNA and contain 64 (4^3^) trinucleotides: AAA, AAC, AAG, …, TTT. Thus, for a sequence with a length of *l*-bp, the detailed information of the trinucleotide positions can be expressed by a 64 × (*l *− 2) matrix *Z*:

6 $$Z = \left[ {\begin{array}{*{20}c} {Z_{1,1} } & \cdots & {Z_{1,l - 2} } \\ \vdots & \ddots & \vdots \\ {Z_{64,1} } & \cdots & {Z_{64,l - 2} } \\ \end{array} } \right],$$

where the variable

7 $$Z_{i,j} = F^{ + } \left( {3{\text{mer}}_{i} |j} \right) - F^{ - } \left( {3{\text{mer}}_{i} |j} \right) \left( {i = 1,2, \ldots ,64; j = 1,2, \ldots l - 2} \right) .$$

*F*^+^(*3*mer_*i*_*|j*) and *F*^−^(*3*mer_*i*_*|j*) denote the frequency of the *i*th trinucleotide (3mer_*i*_) at the *j*th position in the positive and negative datasets, respectively. 3mer_1_ is AAA, 3mer_2_ is AAC,… 3mer_64_ is TTT in Eq. 6.

The sample in Eq. 1 can be expressed as the PSTNP_SS_ vector:

8 $$S = \left[ {\phi_{1} , \phi_{2} ,\phi_{3} ,\phi_{4} , \ldots , \phi_{l - 2, } } \right]^{\text{T}} ,$$

where T is the transpose operator and *ϕ*_*v*_ is defined as:

9 $$\phi_{u} = \left\{ {\begin{array}{*{20}c} {Z_{1,u} ,\,{\text{when}}\;N_{u} N_{u + 1} N_{u + 2} = {\text{AAA}}} \\ {Z_{2,u} ,\,{\text{when }}\;N_{u} N_{u + 1} N_{u + 2} = {\text{AAC}}} \\ {Z_{3,u} ,\,{\text{when}}\;N_{u} N_{u + 1} N_{u + 2} = {\text{AAG}}} \\ \vdots \\ {Z_{64,u} ,\,{\text{when}}\;N_{u} N_{u + 1} N_{u + 2} = {\text{TTT}}} \\ \end{array} } \right. \left( {1 \le u \le l - 2} \right).$$

PSTNP_DS_ features characterize double-stranded position-specified information according to complementary pairing. We deemed A and T as identical, the same to C and G. Each sample could be converted into a sequence containing A and C only. For example, the DNA sequence “TCGAGTGAC” could be converted into “ACCACACAC”. There are only eight (2^3^) trinucleotides: AAA, AAC,…, CCC. Thus, for a sequence whose length is *l*-bp, detailed information on trinucleotide positions can be expressed by an 8 × (*l* − 2) matrix *Z*′:

10 $$Z^{\prime} = \left[ {\begin{array}{*{20}c} {Z^{\prime}_{1,1} } & \cdots & {Z^{\prime}_{1,l - 2} } \\ \vdots & \ddots & \vdots \\ {Z^{\prime}_{8,1} } & \cdots & {Z^{\prime}_{8,l - 2} } \\ \end{array} } \right],$$

where the variable

11 $$Z^{\prime}_{i,j} = F^{ + } \left( {3{\text{mer}}_{i} |j} \right) - F^{ - } \left( {3{\text{mer}}_{i} |j} \right) \left( {i = 1,2, \ldots ,8; j = 1,2, \ldots l - 2} \right).$$

*F*^+^ (*3*mer_*i*_*|j*) and *F*^−^ (*3*mer_*i*_*|j*) denote the frequency of the *i*th trinucleotide (3mer_*i*_) at the *j*th position in the positive and negative datasets, respectively. 3mer_1_ is AAA, 3mer_2_ is AAC, …, 3mer_8_ is CCC in Eq. 10.

The sample in Eq. 1 can be expressed as the PSTNP_DS_ vector:

12 $$S^{\prime} = \left[ {\phi^{\prime}_{1} , \phi^{\prime}_{2} ,\phi^{\prime}_{3} ,\phi^{\prime}_{4} , \ldots , \phi^{\prime}_{l - 2, } } \right]^{\text{T}} ,$$

where *S*′ is the converted sequence and T is the transpose operator. In this formula, *ϕ*′_*v*_ is defined as:

13 $$\phi^{\prime}_{u} = \left\{ {\begin{array}{*{20}c} {Z^{\prime}_{1,u} , \,{\text{when}} N_{u} N_{u + 1} N_{u + 2} = {\text{AAA}}} \\ {Z^{\prime}_{2,u} ,\,{\text{when}} N_{u} N_{u + 1} N_{u + 2} = {\text{AAC}}} \\ {Z^{\prime}_{3,u} ,\,{\text{when}} N_{u} N_{u + 1} N_{u + 2} = {\text{ACA}}} \\ \vdots \\ {Z^{\prime}_{64,u} ,\,{\text{when}} N_{u} N_{u + 1} N_{u + 2} = {\text{CCC}}} \\ \end{array} } \right. \left( {1 \le u \le l - 2} \right).$$

Here, both PSTNP_SS_ and PSTNP_DS_ generated 39 features.

### Physicochemical properties

The pseudo-amino acid composition (PseAAC) method has been successful used to address many computational proteomics problems [44–47] and hastened the application of the pseudo *k*-tuple nucleotide composition (PseKNC) method. In this study, we used a simplified Type-II PseKNC based on physicochemical properties, which can represent the long-range interaction between oligonucleotides. The physicochemical Type-II PseKNC feature was constructed as follows:

14 $${\text{Dp}} = \left[ {d_{1} ,d_{2} ,d_{4} ,d_{4} , \ldots d_{\varLambda } , d_{\varLambda + 1} , \ldots d_{\lambda \varLambda } } \right]^{\text{T}} ,$$

where *d*_*i*_ reflects the long-range sequence-order physicochemical effect of a DNA sequence whose length is *L*-bp and definition is:

15 $$\left\{ {\begin{array}{*{20}l} {d_{1} = \frac{1}{L - k - 1}\sum\nolimits_{i = 1}^{L - k - 1} {J_{i,i + 1}^{1} } } \\ {d_{2} = \frac{1}{L - k - 1}\sum\nolimits_{i = 1}^{L - k - 1} {J_{i,i + 1}^{2} } } \\ {d_{3} = \frac{1}{L - k - 1}\sum\nolimits_{i = 1}^{L - k - 1} {J_{i,i + 1}^{3} } } \\ \vdots \\ {d_{\varLambda } = \frac{1}{L - k - 1}\sum\nolimits_{i = 1}^{L - k - 1} {J_{i,i + 1}^{\varLambda } } } \\ \vdots \\ {d_{\lambda \varLambda - 1} = \frac{1}{L - k - 1}\sum\nolimits_{i = 1}^{L - k - \lambda } {J_{i,i + \lambda }^{\varLambda - 1} } } \\ {d_{\lambda \varLambda } = \frac{1}{L - k - 1}\sum\nolimits_{i = 1}^{L - k - \lambda } {J_{i,i + \lambda }^{\varLambda } } } \\ \end{array} } \right..$$

In Eq. 15, *λ* denotes the tiers or correlation ranks along a DNA sequence and should be set to a signless integer less than *L *− *k*. Λ is the number of physicochemical properties used in feature construction. $J_{i,i + m}^{\psi }$ denotes the correlation of the *ψ*th physicochemical property between the *i*th dinucleotide (*N*_*i*_*N*_*i*+1_) and (*i *+ *m*)th dinucleotide (*N*_*i*+*m*_*N*_*i*+*m*+1_). $J_{i,i + m}^{\psi }$ can be calculated by:

16 $$\left\{ {\begin{array}{*{20}l} {J_{i,i + m}^{\psi } = H_{\psi } \left( {N_{i} N_{i + 1} } \right) \cdot H_{\psi } \left( {N_{i + m} N_{i + m + 1} } \right)} \\ {\psi = 1,2, \ldots ,\varLambda ;m = 1,2, \ldots ,\lambda ;i = 1,2, \ldots ,L - k - \lambda } \\ \end{array} }, \right.$$

where *H*_*ψ*_(*N*_*i*_*N*_*i*+1_) and *H*_*ψ*_(*N*_*i*+*m*_*N*_*i*+*m*+1_) are the values of the *ψ*th physicochemical property for dinucleotides *N*_*i*_*N*_*i*+1_ and *N*_*i*+*m*_*N*_*i*+*m*+1_, respectively. In this study, six double-stranded B-DNA physicochemical properties (e.g., rise, ring, shift, slide, tilt, and twist) from DiProGB (https://diprodb.leibniz-fli.de/ShowTable.php) were used.

Before substituting values into Eq. 16, the original property values were standardized by the formula:

17 $$H_{\psi } \left( {N_{i} N_{i + 1} } \right) = \frac{{H_{\psi }^{0} \left( {N_{i} N_{i + 1} } \right) \cdot \left\langle {H_{\psi }^{0} \left( {N_{i} N_{i + 1} } \right)} \right\rangle }}{{{\text{SD}}\left[ {H_{\psi }^{0} \left( {N_{i} N_{i + 1} } \right)} \right]}},$$

where $H_{\psi }^{0} (N_{i} N_{i + 1} )$ is the original *ψ*th physicochemical property value for *N*_*i*_*N*_*i*+1_ and $\left\langle \bullet \right\rangle$ brackets are the average of quantity therein over the 16 different combinations of A, C, G, and T for *N*_*i*_*N*_*i*+1_. SD is the standard deviation of the corresponding 16 property values.

In this study, *λ* = 5 and there were six (*Λ* = 6) properties. This method generated 30 features.

### Feature selection

Maximum relevance maximum distance (MRMD) [28] was used to select the features. The software package of MRMD was obtained from http://lab.malab.cn/soft/MRMD/index.html.

### Gradient Boosting decision trees

The Gradient Boosting algorithm constructs a strong ensemble learner using multiple weak learners, such as decision trees, and has been applied in a series of biologically supervised classification projects, including prediction of gamma-aminobutyric acid type-A receptors and hot spots at protein–protein interfaces [48, 49]. The Extreme Gradient Boosting (XGBoost) algorithm proposed by Chen and Guestrin [29] is an efficient implementation of Gradient Boosting and has been used extensively by data scientists [50]. The R interface in xgboost v0.81.0.1 was used in this study.

Appropriate tuning of parameters can strengthen a predictor’s discrimination ability. We performed parameter tuning by grid search, with three parameters thus optimized: i.e., maximum tree depth for base weak learners (max_depth, from 2 to 10, step by 1), learning rate (eta, from 0.1 to 0.9, step by 0.05), and gamma (gamma, from 0 to 0.2, step by 0.002). We herein used tenfold cross-validation to select the optimal parameters by accuracy.

### Performance assessment

We used the jackknife test to evaluate the predictor’s performance [51]. Four indices were adopted: i.e., sensitivity (Sn), specificity (Sp), accuracy (Acc), and Matthews Correlation Coefficient (MCC). The indices were defined as:

18 $$\left\{ {\begin{array}{*{20}l} {{\text{Sn}} = 1 - \frac{{N_{ - }^{ + } }}{{N^{ + } }}} \\ {{\text{Sp}} = 1 - \frac{{N_{ + }^{ - } }}{{N^{ - } }}} \\ {{\text{Acc}} = 1 - \frac{{N_{ - }^{ + } + N_{ + }^{ - } }}{{N^{ + } + N^{ - } }}} \\ {{\text{MCC}} = \frac{{1 - \left( {\frac{{N_{ - }^{ + } }}{{N^{ + } }} + \frac{{N_{ + }^{ - } }}{{N^{ - } }}} \right)}}{{\sqrt {\left( {1 + \frac{{N_{ + }^{ - } - N_{ - }^{ + } }}{{N^{ + } }}} \right)\left( {1 + \frac{{N_{ - }^{ + } - N_{ + }^{ - } }}{{N^{ - } }}} \right)} }}} \\ \end{array} } \right.,$$

where $N_{ - }^{ + }$ is the number of positive samples incorrectly predicted to be negative, $N_{{}}^{ + }$ is the total number of positive samples, $N_{ + }^{ - }$ is the number of negative samples incorrectly predicted to be positive, and $N_{{}}^{ - }$ is the total number of negative samples. The four metrics above are valid only for single-label systems.

## Supplementary information

**Additional file 1: Tables S1**, **S2.** Summary of performances of 4 predictors on benchmark dataset and *A. thaliana* dataset.

## Data Availability

The datasets used and analyzed during the current study are available from MethSMRT database (https://sysbio.gzzoc.com/methsmrt) and i6mA-Pred (http://lin-group.cn/server/i6mA-Pred) (Readers can download the *.fasta sequence files of benchmark dataset and *A. thaliana* dataset from https://github.com/Konglab404/p6mA). p6mA was implemented by R language 3.6.1 and the code can also be found at https://github.com/Konglab404/p6mA.

## Abbreviations

XGBoost: Extreme Gradient Boosting
6mA: N^6^-Methyladenine
PSTNP: Position-specific triple-nucleotide propensity
EIIP: Electron–ion interaction pseudopotential
MRMD: Maximum relevance maximum distance
IFS: Incremental feature selection
ROC curve: Receiver operating characteristic curve
auROC: Area under ROC curve
Sn: Sensitivity
Sp: Specificity
Acc: Accuracy
MCC: Matthews Correlation Coefficient
